# Effect of Cultural Priming on Social Behavior and EEG Correlates of Self-Processing

**DOI:** 10.3389/fnbeh.2018.00236

**Published:** 2018-10-08

**Authors:** Gennady G. Knyazev, Ekaterina A. Merkulova, Alexander N. Savostyanov, Andrey V. Bocharov, Alexander E. Saprigyn

**Affiliations:** ^1^Laboratory of Psychophysiology of Individual Differences, Institute of Physiology and Basic Medicine, Novosibirsk, Russia; ^2^Humanitarian Institute, Novosibirsk State University, Novosibirsk, Russia

**Keywords:** collectivism, individualism, priming, social behavior, default-mode network, temporoparietal junction, EEG, connectivity

## Abstract

Humans are social beings and the self is inevitably conceptualized in terms of social environment. The degree to which the self is perceived as fundamentally similar or fundamentally different from other people is modulated by cultural stereotypes, such as collectivism and individualism. These stereotypes are not hardwired in our brains and individuals differ in the degree to which they adopt the attitudes that define their culture. Moreover, individuals can acquire multiple sets of cultural knowledge and, depending on the context, either individualistic or collectivistic cultural mindset could be activated. In this study, we used cultural priming techniques to activate either individualistic or collectivistic mindset and investigated the association between source-level EEG connectivity in the default mode network (DMN) and spontaneous self-related thoughts in the subsequent resting state. Afterward, participants performed a social interaction task, in which they were allowed to choose between friendly, avoidant, or aggressive behavior. After collectivism priming, self-related thoughts were associated with increased connectivity of DMN with the right temporoparietal junction (TPJ), which is involved in taking the perspective of others and is more active in representatives of collectivistic cultures, whereas after individualism priming they were associated with increased connectivity with the temporal pole, which is involved in self/other discrimination and is more active in representatives of individualistic cultures. Individual differences in the intensity of post-priming self-related thoughts and the strength of DMN-temporal pole connectivity predicted individual differences in behavior during the social interaction task, with individualistic mindset predisposing to more friendly and trustful social behavior.

## Introduction

Why in some circumstances other people are treated as friends and allies, whereas in other circumstances they are treated as enemies and aliens? Cultural research indicates that the degree to which other people are perceived as fundamentally similar or fundamentally different from oneself depends on attitudes and values that dominate the culture in which one lives (Yamaguchi, 1994; Triandis, 1995; Hofstede, 2001). Thus, it is generally believed that East Asian cultures emphasize the fundamental relatedness of individuals to each other, whereas Western cultures emphasize the independence from others (Markus and Kitayama, 1991). However, this common view is not always supported by empirical evidence. Thus, in their review of 30 empirical studies, Takano and Osaka (2018) show that the commonly held view that the Japanese are typical collectivists whereas Americans are typical individualists is supported only in 5 studies, whereas 19 studies reported no clear difference, and 11 studies reported that Japanese were more individualistic than Americans. Besides, cultures are not homogeneous in terms of individualism/collectivism. Substantial differences exist within each culture due to geographical restrictions (Realo et al., 1997), or climate (Van de Vliert et al., 2013). Moreover, the relationship between individualism/collectivism and social behavior is not as straightforward as one may expect (i.e., more cooperative and friendly behavior in collectivists and more competitive and egoistic behavior in individualists). At cultural level, individualism correlates positively with personality trait of extraversion, which above all is characterized by sociability and positive emotionality (Hofstede and McCrae, 2004). It has been shown that individualists spend more time with their friends and believe that most people can be trusted (Allik and Realo, 2004). Thus, it is more productive to perceive individualism consisting of three main components: autonomy, mature self-responsibility, and uniqueness (Realo et al., 2002).

Culture-specific attitudes are not hardwired in our brains or genes. People differ in the degree to which they adopt the attitudes that define their culture (Triandis, 1995). Moreover, individuals can acquire multiple sets of cultural knowledge and, depending on the context, either individualistic or collectivistic cultural mindset could be activated (Oyserman and Sorensen, 2009; Oyserman, 2011). Cultural psychologists have developed cultural priming techniques to manipulate cultural value systems within individuals. Meta-analysis of the individualism and collectivism priming literature shows that the results are robust across priming methods and consistent in direction with cross-national effects, which means that depending on situational context human behavior could be switched over between collectivistic and individualistic mindsets (Oyserman and Lee, 2008). For a neuroscientist, the most interesting question is how this switching is implemented in the brain.

Cultural neuroscience has accumulated ample evidence linking culture-related differences in social cognition with differential activity of specific brain circuits. It has been shown for instance that social cognitive processes are accompanied by stronger activity in the dorsal medial prefrontal cortex (MPFC) and temporoparietal junction (TPJ) in East Asians but stronger activity in the ventral MPFC, insula, and temporal pole in Westerners (Han and Ma, 2014). The discovery of the so-called resting state or intrinsic connectivity networks (ICN) has changed the primary focus of interest in the study of human brain function (Biswal et al., 1995). A handful of ICNs revealed in functional magnetic resonance imaging (fMRI) data obtained in rest or in different kinds of tasks are highly functionally relevant (Smith et al., 2009). The so-called default mode network (DMN, Raichle et al., 2001) is the principal network associated with self-referential and social processing (Gusnard et al., 2001; Mitchell, 2006; Gobbini et al., 2007), and existing evidence links this network with culture-related differences in social cognition. In particular, this evidence suggests that collectivistic/individualistic mindset priming is associated with modulations of activity in the DMN (Wang et al., 2013; Oyserman et al., 2014).

One may wonder why a resting state brain activity should be important for behavior. Experimental evidence shows that in some kinds of behavior conscious experiences may come too late to causally affect the behavior (Libet, 1985; Velmans, 1991). It is assumed that for such behaviors the adaptive function of consciousness may be implemented prospectively, which entails a thorough analysis of consequences of past behavior and prospective inhibition or potentiation of future behavioral routines (e.g., Gray, 2004; Corr, 2010). Much of this processing must occur in a resting state. Respectively, it is suggested that the DMN, which is most active in this state, may play a critical role in the organization and expression of preplanned, reflexive behaviors (Raichle, 2015). In this connection, it should be noted that most of the cultural priming research investigated its effect on brain activation during specific experimental manipulations. Its effect on the intrinsic brain connectivity during a resting state is virtually unstudied, apart from the Wang et al.’s (2013) study, who did not find significant difference in synchronization of activities in remote brain regions between different priming conditions. The second gap in the existing evidence is that the overwhelming majority of respective studies used fMRI, whereas EEG was mostly used in the study of attention by means of event-related potential (Oyserman et al., 2014). Although fMRI has excellent spatial resolution, its relation to neuronal events is still a matter of debate (e.g., Debener et al., 2006), which makes electrophysiological confirmation of fMRI findings very important. Recently, an array of methods has been developed for the study of ICNs based on source-level electrophysiological data (de Pasquale et al., 2010; Brookes et al., 2011a,b; Knyazev et al., 2011, 2016b; 2018; Hipp et al., 2012; Siems et al., 2016). To the best of our knowledge, only two studies investigated culture-related effects on electrophysiological correlates of ICNs in a resting state. In one study Knyazev et al. (2012), spontaneous EEG data and retrospective questionnaire measures were obtained in Russian and Taiwanese participants. In both samples, appearance of spontaneous self-referential thoughts was accompanied by enhanced alpha activity within the DMN, which prevailed in the posterior DMN hub in Russian, but in the anterior DMN hub in Taiwanese participants. In another study Knyazev et al. (2018), mediation analysis showed that the relationship between interdependent self-construal and social cognition was mediated by MPFC connectivity with the left middle temporal gyrus in the alpha frequency band.

In this study, we used cultural priming techniques to activate either individualistic or collectivistic mindset in the subsequent resting state. Functional connectivity between the DMN and the rest of the brain was estimated during this state in source-level EEG data filtered in standard frequency bands and correlated with a self-report measure of spontaneous self-related thoughts. Afterward, participants performed a virtual social interaction task, in which they were allowed to choose between friendly, avoidant, or aggressive behavior. We expected that after collectivism, as opposed to individualism priming self-related thoughts would be associated with increased connectivity in brain areas related to social cognition, such as the MPFC and the TPJ. Furthermore, we expected that these effects would be revealed in the alpha frequency band, which is most strongly associated with self-referential processing (for review see Knyazev, 2013). At the behavioral level, we expected that people who think more about the self after individualism priming would show less avoidant and more friendly behavior.

## Materials and Methods

### Participants

Initially the sample included 42 participants. Two participants were excluded from the analysis due to excessive movement artifacts and three participants failed to complete both priming conditions. The remaining sample consisted of 37 Caucasians (mean age = 23.8; *SD* = 6.2; 23 females). Undergraduate and graduate students made up the majority of the sample, others were University staff members. Participants reported no history of neurological or psychiatric disorders, alcohol or drug dependence, or current treatment with vasoactive or psychotropic drugs. All subject protection guidelines were followed in accordance with the Declaration of Helsinki. Each participant signed an informed consent. The study was approved by the Institute of Physiology and Basic Medicine ethical committee.

### Procedure

In order to study the effect of collectivism and individualism priming (hereafter, COLL and IND, respectively), all participants were invited to the laboratory twice. The two visits were 2–3 weeks apart and the order of collectivistic and individualistic priming was random in different participants. Participants were seated in a soundproof dimly illuminated room before a computer screen, which was situated at a distance of 120 cm from the participant. After placing the EEG electrodes, participants were presented with priming instructions, which appeared on the screen: “For the next 2 min, please close your eyes and think of what you have in common with your family and friends” (collectivism priming); or “For the next 2 min, please close your eyes and think of what makes you different from your family and friends” (individualism priming). After the lapse of 2 min, participants via loudspeaker were asked to open the eyes and during the next 12 min just sit patiently and try to minimize movement and blinking. The following procedure consisted of 12 1 min recordings (6 with eyes open and 6 with eyes closed) alternating sequentially. During the eyes open condition, participants were asked to look at the empty computer screen. The first 2 min after the priming were discarded and only the eyes open condition was used in the analysis based on the observation that resting-state connectivity diminishes in the eyes closed, compared to the eyes open condition (Van Dijk et al., 2009). Just after the spontaneous EEG registration participants were asked to fill out the Spontaneous Thoughts Questionnaire (Knyazev et al., 2012), a retrospective measure of subject’s state and thoughts during the EEG registration. All items are scored on a five-point Likert scale. The self-referential thought scale (SRTS) that was used in this study consists of 5 items (example item: “recollected episodes from my own life,” α = 0.72).

Next, we used the social interaction task, which previously proved to be a reliable instrument for the study of social behavior in the laboratory (Knyazev et al., 2011, 2013, 2015, 2016a). As stimulation we used angry, fearful, sad, neutral, and happy facial expressions from the Karolinska Emotional Directed Faces database (Goeleven et al., 2008). The pictures were presented black and white (17 × 17 cm) and displayed on a screen at a distance of 120 cm from the subject. Participants were presented with the instruction, in which they were asked to imagine that faces, which they see at the screen, are living persons and they had to choose one out of three options: “attack,” “avoid,” or “make friends” (pressing “1,” ”2,” or “3” button, respectively). First, a fixation cross appeared at the center of the screen for 1 s. Then a face picture was presented. Angry, afraid, sad, happy, and neutral faces were delivered randomly and inter-stimulus-interval randomly varied between 4 and 7 s. The number of face stimuli was 200 for each participant, including 20 male and 20 female faces of each category. After the experiment, participants filled in a set of psychometric questionnaires and were debriefed.

### EEG Recording

One hundred and eighteen active electrodes mounted in the Quik-Cap128 NSL according to the extended International 10-10 system were used for EEG acquisition. The electrooculogram was recorded simultaneously. Brain Product (Germany) actiCHamp amplifiers with a 0.1–100 Hz analog bandpass filter were used for signal amplification. The sampling rate was 1000 Hz. FASTRAK digitizer (Polhemus) was used to measure the position of each electrode and the three fiduciary points (nasion and two preauricular points). Fronto-central electrode was used as the ground and Cz as the reference. Electrode impedances were kept at or below 5 kilo-ohms. The recordings were first inspected visually and channels with nonstereotyped gross artifacts were removed. Next, artifacts were corrected using independent component analysis and missing channels were interpolated using spherical spline interpolation via the EEGlab toolbox^1^. The average number of interpolated channels was not statistically different in different priming conditions. EEG data were recomputed to the average reference.

### EEG Preprocessing

EEG data were filtered into five frequency bands (delta – 1–4 Hz; theta – 4–8 Hz; alpha – 8–12 Hz; beta – 12–30 Hz, and gamma – 30–45 Hz) using a Butterworth filter and the Matlab’s filtfilt function, which filters the data forward and backward to minimize the phase delays, and down-sampled to 125 Hz.

### Beamforming

The boundary element head model (Fuchs et al., 2001) was used for forward modeling. The cortical mesh of 5124 vertices was obtained from a template Montreal Neurological Institute (MNI) brain. Individual electrode positions were co-registered with the template brain using the three fiduciary points. The linearly constrained minimum variance beamforming (Van Veen et al., 1997) was performed using the SPM-12 toolbox for beamforming (DAiSS)^2^. Covariance matrices were computed using 5 min continuous eyes-open EEG data and regularized using a regularization lambda value of 0.05% of the signal variance averaged over channels (Litvak et al., 2010). The time-series of each source was projected along the dipole direction that explains the most variance, which is equivalent to determining the largest eigenvector and was performed using the singular value decomposition (Ahlfors et al., 2010).

### Seed-Based Correlation Analysis

Due to the ill-posed EEG inverse problem, the source space projections may be artificially interdependent (Schoffelen and Gross, 2009). To correct the signal leakage, we used the orthogonalization of the reconstructed source time-courses with respect to a seed voxel by means of linear regression method (Brookes et al., 2012; Hipp et al., 2012). After leakage correction, the amplitude envelope was calculated as the absolute value of the analytic signal obtained by means of the Hilbert transform. The Hilbert envelope was averaged over 1-s-long windows (Brookes et al., 2011b).

DMN was represented by four seeds: MPFC (-1, 48, -5), posterior cingulate cortex (PCC, -5, -51, 40), and left (LLPC, -45, -71, 35) and right (RLPC, 45, -71, 35) lateral parietal cortex (Fox et al., 2005). For each seed location, a region of interest (ROI) within a 10 mm sphere around the seed was determined and, in each subject separately, correlations were calculated between Hilbert envelope of each ROI’s voxel and the rest of the brain. The voxel with maximal goodness-of-fit index, calculated as the mean z score of all correlations within a DMN mask minus the mean z score of all correlations outside it (Greicius et al., 2004), was selected for seed location in this subject. We used the DMN template, which is described in Smith et al. (2009) and was downloaded from http://www.fmrib.ox.ac.uk/analysis/brainmap+rsns/. After this preliminary procedure, the chosen seeds were used for leakage correction as is described above.

### Statistical Analysis

Pearson correlations between the seed and all other voxels were calculated, Fisher z-transformed, and mean-centered in order to remove between-subject differences in the mean strength of correlations. The obtained connectivity maps were smoothed spatially (FWHM 8 mm) and used for a second-level general linear model analysis in SPM 12. We used full factorial design with two within-subject factors, i.e., priming (collectivism vs. individualism) and seed (four levels). SRTS scores were entered as a second-level covariate, which was allowed to interact with the priming. False positive control was implemented through a combination of voxel-level height threshold (*p* = 0.001) and cluster-level extent threshold (FWE-corrected cluster-level *p* = 0.001). Behavioral data were analyzed in SPSS using repeated-measures ANOVA. Greenhouse-Geisser correction was applied if necessary, but uncorrected degrees of freedom are reported for the sake of clarity.

## Results

### Behavioral Data

There was no significant effect of priming on SRTS scores (*p* > 0.1). Gender did not show significant main effect or interaction with priming in the prediction of SRTS scores (both *p* > 0.1). SRTS scores obtained in the two priming conditions moderately correlated with each other (*r* = 0.68, *p* < 0.001). The social interaction task data were analyzed using repeated-measures ANOVA with three within-subject factors: priming (two levels), face type (five levels), and behavioral choice (three levels). The percent of choices in each combination of these factors was used as the outcome. Participant’s gender was used as a between-subject factor. There was a significant main effect of choice [*F*(2,68) = 52.7, *p* < 0.001], showing that on average participants most frequently chose avoidant and least frequently aggressive behavior, and a face by choice interaction [*F*(8,272) = 39.5, *p* < 0.001], showing that on average participants more frequently attacked angry faces and offered friendship to neutral and happy faces. All other effects were no significant.

### EEG Data

First, the effect of priming on DMN connectivity in the post-priming resting state was tested. In the beta frequency band, the IND > COLL contrast yielded a significant cluster centered in the right middle temporal gyrus (Brodmann area, BA 37) (55, -64, 7; *k* = 7435; *T* = 4.65, cluster p_FWE-corr_ < 0.001). The opposite contrast did not yield significant result at the chosen significance threshold. There were no significant effects in the other frequency bands.

**FIGURE 1:**
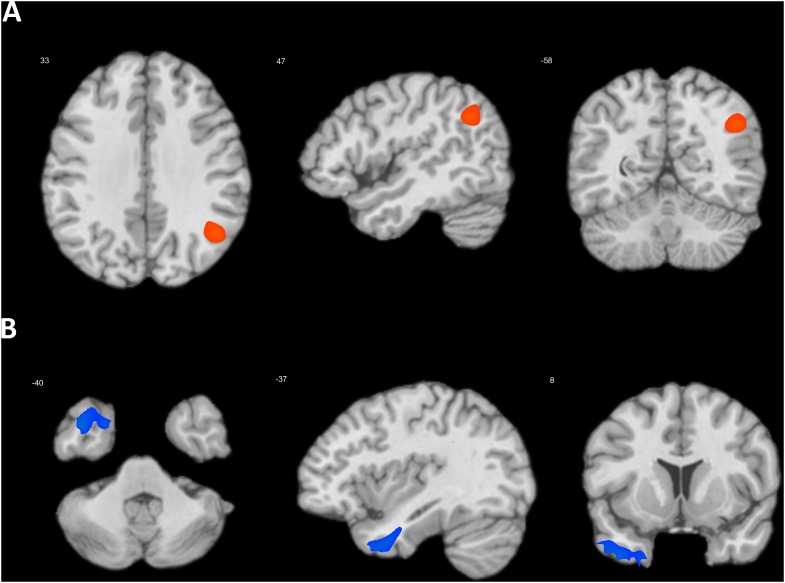
Location of the clusters where the association between DMN connectivity and SRTS scores was higher after collectivism than individualism priming **(A)** and after individualism than collectivism priming **(B)**.

Next, the effect of priming on the association between DMN connectivity and SRTS scores was tested. In the alpha frequency band, the COLL > IND contrast yielded a significant cluster centered in the right TPJ (BA 39) (47, -58, 33; *k* = 8273; *T* = 4.56, cluster p_FWE-corr_ < 0.001) (**Figure 1A**). The opposite contrast showed a cluster centered in the left temporal pole (BA 38), (-37, 8, -40; *k* = 3178; *T* = 4.87, p_FWE-corr_ = 0.001) (**Figure 1B**). There were no significant effects in the other frequency bands. In the *post hoc* analyses, we tested the effect of priming on the association between SRTS scores and the connectivity of each DMN seed separately. The COLL > IND contrast yielded similar results for MPFC (49, -58, 35; *k* = 5903; *T* = 4.22, cluster p_FWE-corr_ < 0.001) and PCC (31, -42, 51; *k* = 4241; *T* = 4.42, cluster p_FWE-corr_ = 0.001) seeds. For LLPC and RLPC the strongest effect was observed in the same area (33, -48, 55 and 31, -46, 43, respectively), but it was not significant (both cluster p_FWE-corr_ > 0.1). For MPFC and PCC seeds, conjunction analysis showed an overlap centered in the right TPJ (48, -58, 33; *k* = 2167; *T* = 4.19, cluster p_FWE-corr_ = 0.01). The IND > COLL contrast yielded significant effect for MPFC (-31, 2, -19; *k* = 5510; *T* = 4.63, cluster p_FWE-corr_ < 0.001) and RLPC (-57, -2, -27; *k* = 1432; *T* = 4.36, cluster p_FWE-corr_ = 0.034) seeds. Conjunction analysis showed marginally significant overlap centered in the left BA 38 (-39, 5, -37; *k* = 863; *T* = 4.03, cluster p_FWE-corr_ = 0.086).

### Effect of SRTS Scores on Behavior

**FIGURE 2:**
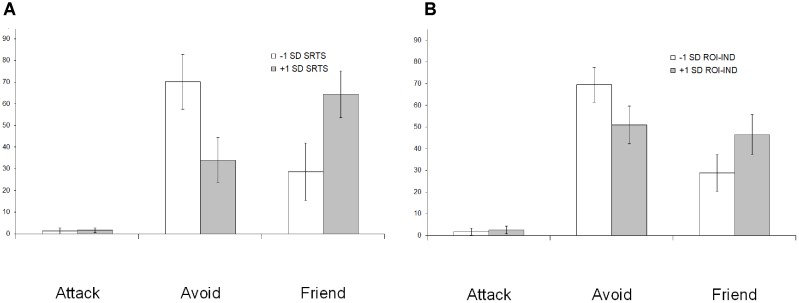
The interaction between behavioral choice in the social interaction task on the one hand, and SRTS scores after individualism priming **(A)** and associated DMN connectivity **(B)** in the preceding resting state on the other hand. Participants who spontaneously thought more about the self after individualism priming and showed higher connectivity between the DMN and the left temporal pole during the resting state (empty bars) less frequently choose avoidant and more frequently choose friendly behavior relative to participants who thought more about the self after collectivism priming and showed higher connectivity between the DMN and the right TPJ (filled bars).

SRTS scores obtained after individualism (SRTS_IND_) and collectivism (SRTS_COLL_) priming, as well as the difference between SRTS_IND_ and SRTS_COLL_ (hereafter SRTS_IND-COLL_) were used as covariates in repeated measures ANOVA of behavioral data. SRTS_COLL_ did not show significant effects. For SRTS_IND_ [*F*(2,68) = 8.4, *p* = 0.001] and SRTS_IND-COLL_ [*F*(2,68) = 10.8, *p* < 0.001] there was a significant interaction with behavioral choice. To uncover the nature of this interaction, we depicted the percentage of behavioral choices in groups of participants falling in the +1 SD (*n* = 7) and the -1 SD (*n* = 7) on SRTS_IND-COLL_ scale. As **Figure 2A** shows, participants with higher SRTS_IND-COLL_ scores less frequently choose avoidance and more frequently choose friendship.

### Effect of DMN Connectivity in the Resting State on Subsequent Behavior in the Social Interaction Task

To investigate the effect of DMN connectivity in the resting state on subsequent behavior in the social interaction task, we extracted average MPFC connectivity values in the alpha frequency band in clusters that showed significant association with SRTS scores in the IND > COLL (ROI_IND_) and COLL > IND (ROI_COLL_) contrasts in the previously described analysis and used them as covariates in repeated measures ANOVA of behavioral data. ROI_COLL_ did not show significant effects. For ROI_IND_, a significant interaction with behavioral choice was revealed [*F*(2, 68) = 5.7, *p* = 0.005]. As **Figure 2B** shows, participants with higher ROI_IND_ scores less frequently choose avoidance and more frequently choose friendship.

## Discussion

In this study, participants were first asked to think of what s/he has in common with other people or what makes her/him different from them. In the subsequent resting state, they were not restricted in their thoughts. The average amount of spontaneous self-related thoughts during this period was not significantly different in the two priming conditions and there was a moderate correlation between SRTS scores obtained in these conditions, implying that some participants have more self-related thoughts than others independently of priming. This correlation, however, explains less than 50% of variance in SRTS scores. A visual inspection of SRTS_IND-COLL_ scores shows that about 50% of participants have negative and the other part of the sample have positive SRTS_IND-COLL_ scores, meaning that some participants had more self-related thoughts after individualism priming, whereas others had more self-related thoughts after collectivism priming. It is reasonable to suggest that the content of these priming-dependent thoughts should be related to the content of respective priming and the analysis of DMN connectivity associated with these thoughts shows significantly different patterns of brain activity.

Collectivism, as opposed to individualism priming caused increased self-processing related connectivity of DMN seeds with the right TPJ. The TPJ, particularly its right counterpart, is involved in taking the perspective of others and inferring their mental states (for reviews, see Saxe, 2006; Van Overwalle and Baetens, 2009). The right TPJ is considered a key node within the “social brain” (Frith and Frith, 2010), which plays a critical role in various aspects of social cognition such as theory of mind (ToM), empathy, and mental state attribution, as well as in social interactions (Decety and Lamm, 2007; Zaitchik et al., 2010; Tang et al., 2016). It is involved in the control of imitation and the ability to switch between representations of the self and other people (Sowden and Catmur, 2015). Depending on parameters, transcranial direct current stimulation of the right TPJ either decreases the accuracy in ToM and cognitive empathy tasks (Mai et al., 2016), or enhances social ability and lie detection (Santiesteban et al., 2012; Sowden et al., 2012). A nexus model for TPJ function suggests that the anatomical convergence of attention, memory, language, and social processing in the TPJ leads to its higher-order role in the creation of a social context for behavior (Carter and Huettel, 2013). Much evidence shows that this part of the DMN is more active in representatives of collectivistic than individualistic cultures (Sul et al., 2012; Han and Ma, 2014, 2015; Han, 2015). Moreover, the measure of interdependent self-construal is positively correlated with TPJ activity and mediation analysis shows that the difference in TPJ activity between representatives of collectivistic and individualistic cultures is fully mediated by this self-construal measure (Ma et al., 2014). Our data indicate that cultural priming may change the pattern of DMN connectivity associated with spontaneous self-referential thoughts even in the same individuals. When priming emphasizes the similarity between the self and other people, thinking about the self is associated with increased connectivity of the right TPJ with other DMN regions, implying that the self in this case is conceptualized taking into account the perspective of others. When, on the other hand, priming emphasizes the difference between the self and others, connectivity between the right TPJ and other DMN parts diminishes and the priority is given to the left temporal pole. Meta-analysis of fMRI data shows a stronger activity of the right temporal pole during social affective processes in Westerners than in East Asians (Han and Ma, 2014). Both counterparts of the temporal pole are involved in social cognitive processes, with the right counterpart being more involved in emotional and the left one in semantic aspects of these processes (Olson et al., 2007; Semenza, 2011). In PET and fMRI studies of perspective-taking, contrasting third-person vs. first person perspective resulted in hemodynamic increase in the left temporal pole, suggesting its role in self/other discrimination (Ruby and Decety, 2004; D’Argembeau et al., 2006; Sugiura et al., 2006). It is important to emphasize that the observed differences in DMN connectivity could not be attributed to a residual effect of the priming itself, because this effect was observed in the beta frequency band, whereas the effect of priming on DMN connectivity associated with self-related thoughts was, as expected, found in the alpha frequency band, in line with much evidence linking alpha oscillations with self-referential mental activity (for review see Knyazev, 2013).

Most interesting finding of this study is that individual differences in the intensity of post-priming self-related thoughts were associated with individual differences in behavior during the social interaction task. Specifically, participants who thought more about his/her self after individualism priming were more inclined to friendly behavior and were less inclined to avoid contact. The same behavioral effect was observed in participants who showed increased connectivity between MPFC and the left temporal pole after individualism priming. These findings imply that the mindset, which emphasizes differences between the self and the closest people and which is associated with a specific pattern of DMN connectivity in the alpha frequency band, predisposes to more trustful social behavior. Paradoxical as this statement may seem, it finds confirmation in the literature. Comparative studies show that on average, people in individualistic cultures are more extraverted, have more friends, and are more tolerant to other people (Triandis, 2000; Hofstede, 2001; McCrae, 2001; Allik and Realo, 2004). Collectivists endorse a sharper differentiation between in-group and out-group members than individualists (Triandis, 2001). People who have closer bonds with their friends and relatives and view themselves similar to them may be less motivated to seek relationships with strangers, whereas people who emphasize their personal uniqueness and independence are more motivated to seek cooperation and friendship with people outside their narrow group (Triandis, 1995; Realo et al., 2002; Allik and Realo, 2004). In this study, the priming instruction prompted to compare the self with close friends and relatives (i.e., in-group members), while in the social interaction task they were confronted with unfamiliar strangers (i.e., out-group members). Hence, the observed effect of individualism vs. collectivism priming on behavior may reflect the inherent to these mindsets distinction between in-group and out-group members.

This study has a number of limitations. The social interaction task is an artificial model of social behavior. Previous studies show that behavior in this task meaningfully correlates with personality and EEG measures of brain activity (Knyazev et al., 2011, 2013, 2015, 2016a), which gives some assurance of its ecological validity, but some aspects of this model only vaguely resemble the real-life social behavior. Thus, the “attack” option is an exaggeration of real-life hostile behavior in the modern society and not surprisingly some participants never choose this option during the experiment. A limitation of the experimental design is that there was a considerable delay between the priming and the social interaction task, which may explain the absence of significant effects of priming itself on the behavior in this task. A limitation of our source localization method is that individual structural MRIs were not available and a template head model was used instead. However, since position of each electrode was measured, the individual head shape and size were taken into account.

In general, results of this study show that cultural priming affects the nature of spontaneous self-related thoughts. This is evident from the fact that after collectivism priming self-related thoughts are associated with increased connectivity of DMN with the right TPJ, which is involved in taking the perspective of others and is more active in representatives of collectivistic cultures, whereas after individualism priming they are associated with increased connectivity of DMN with the temporal pole region, which is involved in self/other discrimination and is more active in representatives of individualistic cultures. Moreover, individual differences in the intensity of post-priming self-related thoughts and the respective pattern of DMN connectivity are associated with individual differences in behavior during the social interaction task, with individualistic mindset predisposing to more friendly and trustful social behavior.

## Author Contributions

GK planned the study, analyzed data, and wrote the initial draft of the paper. EM participated in data analysis. ANS, AB, and, AES collected the data and made preliminary data preprocessing. All authors contributed to the final version of the manuscript.

## Conflict of Interest Statement

The authors declare that the research was conducted in the absence of any commercial or financial relationships that could be construed as a potential conflict of interest.

## Footnotes

1 http://www.sccn.ucsd.edu/eeglab/

2 https://code.google.com/p/spm-beamforming-toolbox/
